# Therapeutic Termination of Pregnancy Under the Umbrella of Environmental, Socio-Economic Factors and High-Risk Pregnancy

**DOI:** 10.3390/diagnostics16070985

**Published:** 2026-03-25

**Authors:** Mihai-Daniel Dinu, Liana Ples, Fernanda-Ecaterina Augustin, Mara-Madalina Mihai, Ancuta-Alina Constantin, Gabriel-Petre Gorecki, Andrei-Sebastian Diaconescu, Mircea-Octavian Poenaru, Romina-Marina Sima

**Affiliations:** 1 Department PhD, IOSUD-Institution Offering Doctoral Studies, “Carol Davila” University of Medicine and Pharmacy, 020021 Bucharest, Romania; mihai-daniel.dinu@drd.umfcd.ro; 2 Department of Obstetrics and Gynecology, “Carol Davila” University of Medicine and Pharmacy, 020021 Bucharest, Romania; liana.ples@umfcd.ro (L.P.); mircea.poenaru@umfcd.ro (M.-O.P.); romina.sima@umfcd.ro (R.-M.S.); 3 “Bucur” Maternity, Saint John Hospital, 012361 Bucharest, Romania; 4 Faculty of Medicine, “Carol Davila” University of Medicine and Pharmacy, 020021 Bucharest, Romania; mara.mihai@umfcd.ro (M.-M.M.); andrei.diaconescu@umfcd.ro (A.-S.D.); 5 Department of Oncologic Dermatology, “Elias” University Emergency Hospital, 010024 Bucharest, Romania; 6 Department of Cardio-Thoracic Pathology, “Carol Davila” University of Medicine and Pharmacy, 020021 Bucharest, Romania; ancuta-alina.constantin@umfcd.ro; 7 Institute of Pneumology “Marius Nasta”, 050159 Bucharest, Romania; 8 Department of Anesthesia and Intensive Care, Faculty of Medicine, “Titu Maiorescu” University, 031593 Bucharest, Romania; gabriel.gorecki@prof.utm.ro; 9 Department of Anesthesia and Intensive Care, CF2 Clinical Hospital, 011464 Bucharest, Romania; 10 General Surgery Department, Fundeni Clinical Institute, 022328 Bucharest, Romania

**Keywords:** therapeutic abortion, congenital anomalies, prenatal screening, prenatal diagnosis, chromosomal abnormalities, psychiatric comorbidities, reproductive rights, abortion ethics

## Abstract

Therapeutic termination of pregnancy (TToP) represents an intervention that is performed for medical reasons, such as risks to maternal health or severe fetal anomalies. Advances in prenatal screening and diagnostic tools—including serum markers, ultrasound, cell-free fetal DNA, chorionic villus sampling and amniocentesis—have significantly improved early detection and clinical decision-making. This narrative review synthesizes current knowledge on the genetic, environmental and psychosocial determinants that influence the decision of the patients to pursue TToP. The literature search was performed primarily using PubMed database, while Scopus and Google Scholar were used to identify additional relevant studies. Some of the selected studies, as well as certain sections of this review, address both therapeutic and voluntary termination of pregnancy, whereas others focus exclusively on TToP. Moreover, this review describes the types of abortion (medical or surgical/aspiration) along with their management strategies to prevent or address potential complications. It is well known that demographic, cultural and socio-economic factors continue to influence the access to TToP, as well as the perceptions of it. Psychiatric comorbidities (such as anxiety, affective and psychotic disorders) are observed with a higher prevalence among women undergoing TToP and may influence both the decision and psychological outcomes post-procedure. While most women report emotional relief after TToP, some of them experience depression, post-traumatic stress disorder or substance misuse. Legal and ethical considerations further complicate access to safe abortion, leading to situations where patients may resort to unsafe procedures, which result in higher rates of morbidity and mortality. Data from the EUROCAT network show rising trends in congenital anomalies like trisomy 13, trisomy 18 and caudal regression syndrome (conditions commonly associated with TToP). Therefore, it is mandatory to form a multidisciplinary team in these cases, integrating medical, psychological and ethical dimensions. Ensuring safe, evidence-based and compassionate access to TToP remains a critical component of reproductive healthcare.

## Simple Summary

Therapeutic abortion is a procedure that is performed in well-established medical situations. The aim of this review is to explore medical, psychological, social, and legal aspects regarding the decision to undergo the interruption of pregnancy. Furthermore, we considered it necessary to describe the current diagnostic tools and to outline the key risk factors (such as maternal age, genetic conditions and psychiatric disorders). The importance of counseling, awareness of ethical dilemmas and safe access to care is emphasized, especially in countries with restrictive abortion laws. Therefore, it is important to understand these factors in order to ensure the necessary care and support for women facing this complex situation.

## 1. Introduction

Therapeutic termination of pregnancy (TToP) is a procedure of induced abortion for medical reasons (either to prevent significant harm to the mother or due to fetal unviability). In many jurisdictions, legal thresholds for TToP differ according to gestational age and the maternal or fetal indication [1,2]. Unlike voluntary termination of pregnancy (VToP), which is based on the individual’s choice, TToP requires a physician’s clinical evaluation, making it both a traumatic obstetric event and a procedure with an inherent therapeutic purpose. The decision of whether to terminate a pregnancy, either voluntarily or therapeutically, is a very personal one and is determined by many factors, while broader socio-cultural and economic conditions impact attitudes toward family planning, contraceptive use and abortion. For example, during the COVID-19 pandemic, therapeutic abortion was considered in specific cases of severe respiratory failure. Preterm delivery, as well as TToP, were suggested as possible options to reduce intra-abdominal pressure in pregnant women requiring mechanical ventilation. Furthermore, continuation of pregnancy could limit the use of antiviral or immunomodulatory therapies, given that their fetal safety profiles were not fully established [3,4,5].

Intrauterine development can be either normal or abnormal, with disruptions caused by genetic disorders, environmental factors or a combination of both. These disturbances affect cytogenesis, histogenesis as well as morphogenesis, resulting in congenital anomalies (CAs) at birth [6]. Accounting for a significant proportion of prenatal and neonatal deaths worldwide, CAs are a major global childhood health issue, with prevalence rates ranging from 39.7 per 1000 live births in high-income regions to 82 per 1000 in low-income areas [7]. In many cases, the affected infants do not survive, with more than 70% of them dying within the first month of life. However, even when survival is possible, the treatment is often expensive and full recovery is not always achievable [8].

Although the exact causes of various CAs remain unknown (40–60%), genetic abnormalities, environmental influences and multifactorial inheritance are recognized as key risk factors. Genetic disorders stem from chromosomal abnormalities (which manifest as numerical or structural changes) or single-gene mutations, both contributing to birth defects [6,8].

Birth defects and genetic disorders affect approximately 3% to 5% of pregnancies and are considered a significant cause of infant and childhood mortality [9]. Chromosomal abnormalities (including structural or numerical alterations such as aneuploidy, translocations, duplications and deletions) occur in 1/150 live births. Trisomy 21 (Down syndrome) stands as the most prevalent chromosomal disorder with a birth incidence of 1 in 800, while trisomy 13 and trisomy 18 occur less frequently (but are often associated with elevated infant mortality rates). Sex chromosome aneuploidies are rarer than autosomal aneuploidies. Monosomy X (known as Turner syndrome) is the only monosomy compatible with life [10].

The risk of aneuploidy increases with maternal age, but other factors, such as ultrasound findings, past obstetric history, and familial genetic conditions, also play a role [10]. It was observed that the likelihood of recurrence increases if there was a previous pregnancy affected by aneuploidy or another genetic disorder, especially if one of the parents is a carrier of a balanced Robertsonian translocation. However, the majority of cases are sporadic due to chromosomal nondisjunction.

The identification and prevention of environmental causes of congenital anomalies is crucial. Approximately 10% of these anomalies are attributed to such factors and may be prevented through appropriate interventions. Teratogens that affect the human embryo during critical phases of development can cause malformations by disrupting normal development (particularly during rapid organ differentiation) [8,11,12]. Potential risk factors include maternal infections, advanced maternal age, drug intake and exposure to substances (such as caffeine, nicotine, commonly used medications and hazardous waste). Some studies have also described that maternal nutritional status, alcohol consumption, obesity, diabetes, parental race, socio-economic status and hyperthermia during early pregnancy are all linked to congenital anomalies (CAs) [6]. Certain chemical substances, including mercury, lead and arsenic, also contribute to congenital abnormalities. It has been shown that mercury exposure can lead to neurological impairments resembling cerebral palsy. Moreover, lead was linked to fetal growth restriction and neurological disorders [6,12]. Furthermore, radiation exposure (e.g., X-rays), depending on dose and timing, may lead to congenital malformations such as spina bifida, cleft palate, limb abnormalities, visual impairment, or microcephaly [8].

Multifactorial inheritance, involving complex gene–gene and gene–environment interactions, plays a significant role in congenital malformations [11,13]. Some studies using mouse models of neural tube defects have demonstrated the impact of these interactions on the development of CAs [11].

## 2. Materials and Methods

This narrative review was conducted to synthesize the current knowledge on the genetic, environmental and psychosocial determinants that influence the decision of patients to pursue therapeutic termination of pregnancy (TToP). Furthermore, this review aimed to describe the available prenatal screening and diagnostic tools—including serum markers, ultrasound, cell-free fetal DNA (cfDNA), chorionic villus sampling (CVS) and amniocentesis—the types of abortion procedures (medical and surgical/aspiration) along with their management strategies, the role of psychiatric comorbidities in the decision-making process and post-procedure outcomes, as well as the ethical, religious and legal considerations surrounding TToP.

The literature search was performed primarily using the PubMed database, while Scopus and Google Scholar were used to identify additional relevant studies. The search strategy included the following key terms, used individually and in combination: “therapeutic abortion”, “therapeutic termination of pregnancy”, “congenital anomalies”, “prenatal screening”, “prenatal diagnosis”, “chromosomal abnormalities”, “psychiatric comorbidities”, “reproductive rights” and “abortion ethics”. Boolean operators (AND, OR) were employed to refine the search results. No restrictions were applied regarding the publication date. The majority of the included references were published between 2010 and 2025, although a limited number of earlier seminal studies were also considered where relevant. Priority was given to original research articles, systematic reviews and clinical practice guidelines published in peer-reviewed journals. Reference lists of key articles were also screened to identify further pertinent publications.

It should be noted that some of the selected studies, as well as certain sections of this review, address both therapeutic and voluntary termination of pregnancy (VToP), whereas others focus exclusively on TToP. Studies were included if they provided relevant data on the medical, psychological, sociodemographic or legal aspects of pregnancy termination. The types of included studies comprised observational studies (cross-sectional, cohort and case–control designs), randomized controlled trials, systematic reviews and meta-analyses, clinical practice guidelines (e.g., ACOG Practice Bulletins), and epidemiological surveillance reports (e.g., EUROCAT/JRC reports). Editorials, commentaries and non-peer-reviewed sources were excluded. The findings were organized thematically into the following categories: prenatal screening and diagnostic methods, sociodemographic and reproductive factors influencing TToP, psychopathological determinants and outcomes, medical and surgical methods of termination, epidemiological trends from the EUROCAT network, as well as ethical, religious and legal considerations.

## 3. Screening and Diagnostic Testing

Women may seek prenatal screening or diagnostic testing for multiple reasons, such as the consideration of pregnancy termination in cases where severe fetal anomalies are detected. The early detection of such anomalies enables parents to make informed decisions, which may include opting for medical interventions, planning for specialized care or choosing TToP if the condition is incompatible with life (or significantly affects the quality of life) [14]. It has been observed that neural tube defects can be treated prenatally, which may lead to better neonatal outcomes. The ethical and emotional nature of these decisions requires that patients receive detailed counseling about the risks, benefits and limitations of the tests that are available. Appropriate guidance, aligned with their values and medical needs, should be offered by genetic counselors and healthcare providers.

### 3.1. Screening Tests

Prenatal testing is primarily conducted as a screening tool to assess the likelihood of chromosomal abnormalities or birth defects. The testing methods used for prenatal screening include serum screening, carrier screening and ultrasound. The ultrasound test delivers precise diagnoses for open neural tube defects, but serum screening generates only probability results. Various serum screening options exist, differing in testing criteria and timing [10].

#### 3.1.1. First-Trimester Screening

The first-trimester screening is conducted between 10 weeks and 13 weeks, 6 days of gestation. This screening test is used to assess the risk of chromosomal abnormalities through serum marker analysis combined with ultrasound evaluation. The serum test measures free beta-human chorionic gonadotropin (hCG) and pregnancy-associated plasma protein A (both markers are collected during the 9 to 13 weeks, 6-day period). This data, along with maternal age, prior pregnancy history, fetal count, weight, race and nuchal translucency measurement (NT), is used to estimate the risk (with some models also considering the presence of the nasal bone). The risk is expressed as a ratio, with 1 in 300 often considered the threshold for high risk, although this varies by laboratory. The detection rate for trisomy 21 ranges from 82% to 87%, with a 5% false-positive rate [10,15,16].

Pregnant individuals who have an enlarged nuchal translucency need to undergo targeted ultrasound and fetal echocardiography regardless of their aneuploidy status [10].

The primary advantage of first-trimester screening is the early availability of results, enabling further diagnostic testing, genetic counseling, and timely consideration of TToP (in severe or lethal cases). The measurement accuracy requires trained sonographers because measurement variations as small as 0.5 mm can significantly affect sensitivity [17]. The detection ability of this test exceeds the quadruple marker screen, but its performance weakens after 13 weeks of gestation (with maximum success at 11 weeks of pregnancy) [18].

Table 1 summarizes major structural anomalies that may be identified during the first trimester scan, along with their diagnostic features and clinical implications.

#### 3.1.2. Quadruple Marker Screening

The quadruple marker screen (quad screen) was introduced in the 1990s and remains widely used for patients who seek care after the first trimester (serving more than 25% of patients in public health clinics). This test is performed between 15 and 22 weeks of pregnancy and measures human chorionic gonadotropin (hCG), alpha-fetoprotein (AFP), inhibin A and unconjugated estriol. It estimates the risk of chromosomal abnormalities by combining serum marker results with maternal factors such as age, race, weight, fetal count, diabetes status and gestational age. The quad screen detects approximately 81% of trisomy 21 cases with a 5% false-positive rate, making its detection rate slightly lower than that of first-trimester screening [10].

A key advantage of the quad screen is its ability to detect open neural tube defects through AFP levels, as this protein is produced by the fetus and can be detected in both amniotic fluid and maternal serum. The quad test offers better accessibility because it does not need specialized sonography equipment, which makes it available in various healthcare facilities. Variations of this test exist, such as the triple screen (which excludes inhibin A) [52] and the penta screen (which incorporates hyperglycosylated hCG) [53]. However, these alternatives do not significantly improve diagnostic performance.

#### 3.1.3. Cell-Free Fetal DNA Screening

Non-invasive prenatal screening (NIPS) using cell-free DNA (cfDNA) has revolutionized aneuploidy detection since 2011. The analysis of fetal DNA fragments in maternal blood requires a fetal fraction above 4% to generate reliable results. The detection rate for trisomy 21 reaches 99%, though the accuracy for trisomies 18 and 13 is slightly lower [54,55].

Despite its high sensitivity, cfDNA remains a screening tool rather than a diagnostic test. The positive predictive value (PPV) of aneuploidy tests depends on the prevalence of aneuploidy in the population, so confirmatory testing with chorionic villus sampling (CVS) or amniocentesis is required before TToP. Cases of premature pregnancy termination without diagnostic confirmation have been reported due to misinterpretation, highlighting the need for genetic counseling and ultrasound evaluation prior to testing [56,57].

Beyond aneuploidy, cfDNA can determine fetal sex, Rh status, and, in some cases, microdeletions and other aneuploidies. However, these additional tests remain unvalidated and have a low PPV. The following conditions may produce false-positive results in cfDNA tests: maternal chromosomal abnormalities, confined placental mosaicism, malignancies and vanishing twin syndrome [58,59]. While cfDNA is the most sensitive test for trisomy 21, traditional sequential screening remains superior for detecting a broader range of chromosomal abnormalities.

#### 3.1.4. Ultrasound Screening

During prenatal care, ultrasound is commonly used to determine gestational age and detect structural abnormalities in the fetus. Many patients opt for ultrasound-only screening, especially second-trimester transabdominal ultrasonography (18–23 weeks), to assess fetal anatomy. First-trimester ultrasounds (whether transvaginal or transabdominal) help confirm viability, detect multiple gestations and identify major anomalies (like anencephaly or cystic hygroma), which are associated with chromosomal abnormalities [10].

The anomalies detectable by ultrasound in the first trimester have already been discussed. Table 2 presents the key anomalies that can be identified during the second trimester. The detection of these anomalies further refines prognostic assessment and supports multidisciplinary counseling, with TToP remaining an important option in cases of severe or complex fetal conditions.

### 3.2. Diagnostic Testing

Diagnostic testing options such as chorionic villus sampling (CVS) and amniocentesis should be discussed and offered to all pregnant patients, particularly when confirmatory testing is desired after screening or ultrasound findings. However, these procedures carry potential risks [88].

#### 3.2.1. Chorionic Villus Sampling

CVS, performed between 10 and 14 weeks, allows for early genetic diagnosis via fluorescence in situ hybridization (FISH), karyotyping, microarray and molecular analysis. Medical professionals perform CVS through transcervical or transabdominal procedures to extract placental tissue while using ultrasound guidance. The procedure allows for early prenatal diagnosis and provides safer and earlier pregnancy termination when needed. However, 1–2% of cases may exhibit confined placental mosaicism, which may affect fetal growth [89]. Pregnancy loss due to CVS is approximately 1 in 455 [88,90].

#### 3.2.2. Amniocentesis

The procedure of amniocentesis, which takes place after 15 weeks, involves sampling amniotic fluid to assess genetic disorders as well as fetal infections and neural tube defects. Amniocentesis provides better detection of confined placental mosaicism than CVS. However, when performed in earlier gestations, it is associated with a higher risk of complications. The procedure carries a pregnancy loss rate of approximately 1 in 900 cases [88,90].

#### 3.2.3. Cytogenetic Analysis

The most reliable method for detecting chromosomal abnormalities involves chromosome analysis from CVS and amniocentesis samples. The clinical use of cytogenetic testing faces certain complexities which require proper evaluation of test outcomes.

Mosaicism occurs when an individual has two or more genetically distinct cell populations. True mosaicism requires multiple independent cultures to reveal the same findings, whereas pseudomosaicism represents single-culture occurrences that usually stem from in vitro artifacts, and is generally not clinically relevant. The occurrence of confined placental mosaicism (CPM) affects about 1% to 2% of pregnancies and is restricted to placental tissue, which may lead to discrepancies between placental and fetal karyotypes [91]. This can result in false-positive cfDNA results, making amniocentesis preferable over CVS when confirmatory testing is required. In some trisomies, particularly trisomy 15, a diploid fetus may arise through trisomy rescue, which carries a risk of uniparental disomy and potential imprinting disorders (such as Prader–Willi and Angelman syndromes). Additionally, it has been observed that CVS has a slightly higher risk of cell culture failure compared to amniocentesis, though this remains rare.

A variety of cytogenetic techniques are available, each offering different levels of resolution. Karyotyping shows effectiveness in detecting large chromosomal deletions or duplications that exceed 5 million base pairs, while chromosomal microarray analysis (CMA) detects copy number variations down to 50,000 base pairs [91]. The FISH technique is used by healthcare professionals for a rapid detection of common autosomal aneuploidies and selected microdeletion syndromes (such as DiGeorge syndrome). Single-gene disorders require targeted molecular testing to confirm the presence or absence of pathogenic mutations.

Given that aneuploidy detection is the primary indication for invasive testing, FISH is often utilized as the first diagnostic test because it delivers results within 48 h without needing cell culture. Despite its diagnostic capability, FISH results should be confirmed by karyotyping due to the possibility of false positives and false negatives [92]. Microarray testing (which can be performed on uncultured cells) has become a preferred method due to its ability to detect both aneuploidy and smaller chromosomal imbalances with faster turnaround times. Additionally, the CMA test works with nonviable cells which makes it especially useful for examining fetal demise or stillbirth samples. Due to these advantages, microarray testing is now recommended as the first-line diagnostic approach for structural anomalies, along with FISH, replacing conventional karyotyping in many cases [88].

With the increasing implementation of CMA, a growing number of chromosomal variants of uncertain significance are being detected, which can contribute to parental anxiety. When a chromosomal variant of uncertain significance appears during testing, the parents usually undergo genetic evaluation to establish inheritance of the variant. A parent’s presence of the variant typically indicates it is harmless. The occurrence of chromosomal variants of uncertain significance in structurally normal pregnancies reaches 1.7%, which demonstrates the necessity of complete pre-test counseling to address potential uncertainties and guide parental decision-making [88,93].

#### 3.2.4. Preimplantation Genetic Diagnosis (PGD)

PGD, performed after in vitro fertilization (IVF), allows healthcare providers to detect chromosomal abnormalities through polar body or blastocyst cell analysis before embryo transfer. Despite its potential, confirmatory CVS or amniocentesis is recommended due to the fact that false-negative PGD results may occur [94]. The negative predictive value of normal FISH results in PGD testing stands at 81% according to research findings [95]. While PGD has minimal risk beyond cost [88], it represents an advancing field in prenatal genetic testing.

## 4. Sociodemographic and Reproductive Factors Influencing TToP/VToP

It is well known that a range of socio-cultural, economic, and demographic factors influence women’s choices regarding pregnancy termination, whether due to an unplanned pregnancy or maternal health concerns. However, many studies fail to distinguish between therapeutic and elective terminations, which can yield biased conclusions about the decision-making processes for TToP. Table 3 summarizes key sociodemographic factors and their impact on abortion rates.

## 5. Psychopathological Determinants of TToP

Various studies have examined the mental health consequences of TToP, often overlooking pre-existing mental health conditions that could affect outcomes. According to the literature data, women who have undergone TToP or VToP generally show higher levels of psychopathology before the procedure compared to those who give birth, and they often seek psychiatric help both before and after the abortion [113,114,115,116]. The majority of mental health problems appear before abortion instead of developing because of it [117]. Women with a history of psychiatric disorders (such as anxiety, mood disorders) or personality disorders are at a higher risk of undergoing abortion. Research indicates that women who underwent abortion procedures developed axis I disorders in 68.3% of cases, while this condition occurred in only 42.2% of women who did not have an abortion [118]. These women are often more likely to experience unwanted pregnancies, engage in risky behaviors, and have a greater desire for abortion due to mental health challenges and socio-economic factors.

Table 4 outlines major psychiatric determinants and their implications for pregnancy outcomes.

## 6. Psychopathological Outcomes After TToP

A medically induced abortion is considered to have a high impact on a woman’s mental and reproductive health, as pregnancy termination is often a traumatic event for parents. The experience of induced abortion differs between the pregnant woman and the baby’s father, with the woman’s response influenced by her desire to give birth and the timing of the procedure [136].

According to Fergusson et al. [136], second-trimester therapeutic termination of pregnancy (TToP) has more psychological consequences and has higher prevalence of psychiatric disorders as opposed to voluntary termination of pregnancy (VToP) or miscarriage that takes place earlier in the pregnancy. Some of the possible reasons could be related to the following factors:

- Surgical Procedure—TToP requires a surgical procedure, unlike VToP or medical management of miscarriage, contributing to a greater psychological burden [136].
- Parental Bonding—Parents undergoing TToP may have already bonded with the fetus and adapted to the parental role, making the psychological impact greater [136].
- Maternal–fetal Attachment—In the first trimester, the absence of fetal movement results in a weaker maternal–fetal attachment, which may lessen the emotional impact compared to TToP [136].
- Post-abortion Distress—After TToP, parents may hold and photograph the baby, intensifying emotional distress, which is less common in VToP or miscarriage [136,137,138].

However, TToP has some positive psychological effects, especially for women who have high distress related to fetal abnormality. Research has shown that TToP can reduce pre-abortion psychological distress (especially regarding mood and anxiety symptoms). Research has shown that depressive and anxiety symptoms tend to decrease in the weeks following the procedure, with long-term mood stabilization. Careful evaluation of individual circumstances can help predict mental health outcomes and guide appropriate psychological support [138,139,140,141,142].

In this context, peri-procedural emotional support remains essential, particularly in second-trimester TToP [136]. The presence of trained staff, respectful communication and ensured privacy represent key factors that may reduce acute distress. Moreover, post-procedural follow-up should include psychological evaluation, especially in patients with identified risk factors (e.g., pre-existing psychiatric disorders, limited social support, or coercion Grief counseling, mental health referral and contraceptive counseling are recommended to reduce long-term psychological morbidity and prevent repeat unintended pregnancies. Counseling and psychological support during the peri- and post-procedural phases of TToP should be considered integral components of standard care [105,139,143,144,145,146,147].

Table 5 presents the most common mental health outcomes and associated risk factors.

A wide range of factors can shape the psychological aftermath of a therapeutic abortion. Table 6 details individual, social and clinical elements that may predict adverse outcomes.

## 7. Medical and Surgical Methods of TToP/VToP

It is known that termination of pregnancy can be performed via two primary approaches: medical (pharmacologic) and surgical (instrumental or aspiration). The choice depends on gestational age, clinical indications, patient preference, and local legal and resource constraints [156,157,158]. Both medical and surgical abortion methods are safe and effective when performed according to evidence-based guidelines.

### 7.1. Pre-Abortion Evaluation

A thorough pre-abortion evaluation is required before initiating any abortion method. This step typically consists of the following: medical history, allergy assessment, confirmation of gestational age (by last menstrual period or ultrasound), pelvic ultrasound to confirm intrauterine pregnancy, and specific laboratory tests (e.g., complete blood count, Rh status, glucose levels in diabetic patients, coagulation profile if anticoagulants are being taken) [159,160]. Patients are counseled regarding options, risks and follow-up. Informed consent is essential.

In addition to medical evaluation, well-structured pre-procedural counseling is necessary and should be considered an essential component of care. This includes providing clear information regarding diagnosis, prognosis, available options (continuation of pregnancy versus TToP), as well as potential physical and psychological outcomes. Psychological assessment usually consists of identifying pre-existing psychiatric conditions, levels of distress, decisional conflict and social support. When necessary, it is recommended to refer to a mental health professional or genetic counselor [102,117,118,136,148,149].

### 7.2. Medical Abortion

Medical abortion is recommended up to 11 weeks of gestation. It may be performed at home or in clinical settings. It is non-invasive and is considered to provide a greater sense of privacy and autonomy [161,162,163]. The preferred regimen for medical abortion is mifepristone followed by misoprostol, while methotrexate is used rarely. The standard protocol includes administration of mifepristone (taken as a single 200 mg oral tablet, typically on Day 1) followed by misoprostol, which is administered 24–48 h later in one of the following routes:

- Buccal: Four 200 mcg tablets placed between the cheek and gum for 30 min, then swallowed.
- Vaginal: Four 200 mcg tablets inserted into the vagina 6 to 48 h after mifepristone.
- Sublingual: Two to four 200 mcg tablets are placed under the tongue for 30 min [161,162,163].

According to the NAF 2020 guidelines, if the patient is more than 63 days from the last menstrual period (LMP), a second dose of 800 mcg misoprostol can be administered four hours after the first dose. For pregnancies beyond 70 days, the second dose of misoprostol is recommended four hours after the administration of mifepristone [161,162,163].

NSAIDs are typically sufficient for pain management during medical abortion at home; routine opioid prescription is not necessary, but a short course may be offered when NSAIDs are contraindicated or not tolerated [164]. The use of prophylactic antibiotics is usually not recommended. Contraceptive counseling can be offered during follow-up, depending on the patient’s interest.

Patients must be advised to contact their healthcare provider [161,165] if they experience the following:

- Excessive bleeding (e.g., soaking two or more pads in two consecutive hours).
- Severe pain that is unresponsive to analgesics.
- Fever > 38 °C (100.4 °F) that lasts for more than 24 h after misoprostol.
- No bleeding within 24 h of misoprostol administration.
- Persistent nausea, vomiting, diarrhea, or abdominal pain beyond 24 h.

Follow-up to confirm abortion success does not always require ultrasound. According to the NAF 2020 guidelines, clinical history and home pregnancy tests may be sufficient [166]. Alternatively, quantitative hCG testing can be performed: it is considered that a decrease of ≥50% by 72 h, ≥60% by 4–5 days, and ≥80% by day 7 from mifepristone administration is consistent with successful abortion [167,168]. Ultrasound remains a valid option for confirming expulsion by demonstrating the absence of a gestational sac or embryo.

In rare cases, a regimen using methotrexate and misoprostol may be employed [161,162,163]. The evidence-based protocol includes oral or intramuscular methotrexate followed three to five days later by vaginal misoprostol for gestational ages up to 63 days.

Contraindications to medical abortion include the following: the presence of an intrauterine device (IUD, which should be removed prior to the procedure); known allergy to mifepristone or misoprostol; and chronic adrenal insufficiency (particularly in patients undergoing long-term systemic corticosteroid therapy). Other medical conditions that preclude medication abortion include suspected or confirmed ectopic pregnancy, hemorrhagic disorders, anticoagulant therapy (excluding low-dose aspirin), hemodynamic instability, and inherited porphyria. On the other hand, conditions such as anemia, seizure disorders, asthma managed with steroid inhalers, obesity, breastfeeding, HIV/AIDS and other sexually transmitted infections are not considered contraindications [161].

Complications of medical abortion may occur despite high safety and efficacy rates, particularly when gestational age is underestimated or patient selection criteria are not strictly followed. Management strategies must be timely and based on clinical presentation [161,165]. Table 7 describes the complications of medical abortion and the therapeutic interventions required for them.

### 7.3. Surgical or Aspiration Abortion

Surgical abortion is typically used up to 16 weeks via manual or electric vacuum aspiration. After 16 weeks, dilation and evacuation (D&E) is indicated and must be performed by experienced clinicians. Aspiration is brief (5–10 min) and performed in a clinic or hospital under local anesthesia, with or without sedation [171].

Instrument kits may include manual vacuum aspirator, cannula, syringe, tenaculum, speculum, cervical dilators, forceps and gauze. In D&E procedures, additional specialized tools and real-time ultrasound guidance are essential in order to ensure safety and effectiveness. In cases of advanced gestation, induced fetal demise may be required before the procedure [172,173].

Steps include bimanual examination, cervical dilation with mechanical or pharmacological agents—such as misoprostol or osmotic dilators (e.g., Dilapan, laminaria)—antiseptic cervical preparation, insertion of the cannula, and aspiration of uterine contents. Osmotic dilators may be placed in the cervix in advance to ensure adequate dilation, particularly in second-trimester procedures. Misoprostol and mifepristone can also aid in cervical ripening. If molar pregnancy is suspected, the aspirated tissue should be sent to pathology for histological confirmation [172,173].

Ultrasound may be employed during or after the procedure to verify completion. Uterotonics are routinely used to manage uterine tone and minimize bleeding (both intra- and post-procedurally). Prophylactic antibiotics are usually administered to reduce the risk of post-procedural infections [174,175]. A summary of complications and management strategies for aspiration abortion is provided in Table 8.

## 8. The EUROCAT Network

Congenital anomalies are a leading cause of infant mortality and morbidity worldwide. The EUROCAT network, established in 1979, monitors these anomalies across 21 countries, covering approximately a quarter of Europe’s birth population. Since 2015, its Central Registry has been managed by the European Commission’s Joint Research Center (JRC) as part of the European Platform on Rare Disease Registration. With data from over 1.1 million births annually, EUROCAT collects epidemiological information, identifies teratogenic risks and evaluates prevention measures [176,177,178].

Each year, the JRC-EUROCAT Central Registry analyzes data from live births, fetal deaths (≥20 weeks gestation) and terminations of pregnancy for fetal anomalies (TOPFA). This allows for trend monitoring and the detection of temporal clusters (which may indicate teratogenic exposures). When unusual case clusters are identified, local registries conduct investigations and notify public health authorities for timely intervention [176,177,179,180].

The pan-European trend analysis monitors prevalence rates for 94 congenital anomaly subgroups, particularly those with insufficient case numbers for local-level evaluation. For the 2013–2022 period, the following trends were identified:

### 8.1. Increasing Trends Identified at Pan-European Level

EUROCAT surveillance has revealed several congenital anomalies with rising prevalence over the past decade. Table 9 highlights those conditions and discusses potential contributing factors.

While these trends may be partially explained by advances in prenatal diagnostics and changes in coding practices, maternal factors (such as obesity or maternal diabetes) may also contribute. Each condition is described below with clinical details and its potential implications for therapeutic abortion decisions.

Double-Outlet Right Ventricle (DORV)—DORV is described in the literature as a serious congenital heart defect in which both the aorta and pulmonary artery arise from the right ventricle [185]. This condition is usually diagnosed early. While some infants may initially be asymptomatic, corrective surgery is often required within the first year of life. In severe cases where surgical intervention is not successful or where significant complications arise (such as severe cyanosis or heart failure), therapeutic abortion may be considered. The rate of survival with early surgery is considered to be high. On the other hand, if DORV is left untreated, the severity of the disease can lead to a poor quality of life, making abortion a potential option when no corrective measures are available.

Caudal Regression Sequence (CRS)—CRS, a rare and severe defect of the sacrum and lumbar spine, is often linked to maternal pre-gestational diabetes. This condition can result in major disabilities, such as paralysis, bowel and bladder dysfunction and a limited lifespan [186,187]. In severe cases, where the child may be born with a severe form of malformed spine as well as potential associated anomalies (e.g., renal malformations, absence of lower limbs), therapeutic abortion is often considered due to extreme disabilities and poor prognosis. CRS cases diagnosed early in pregnancy offer an opportunity for parents to consider the potential long-term suffering and the available options for care.

Patau Syndrome (Trisomy 13) and Edward’s Syndrome (Trisomy 18)—Both Patau Syndrome and Edward’s Syndrome are chromosomal disorders that result in severe intellectual disabilities, profound developmental delays and early death (often within the first year of life) [188,189,190]. These conditions generally lead to death in infancy, and the decision for therapeutic abortion is often made early in pregnancy based on the diagnosis (as these syndromes have no effective treatment options and are associated with immense suffering for the child). For some parents, abortion is seen as a relevant choice given the certainty of a very short and painful life for the infant.

### 8.2. Decreasing Trends Identified at Pan-European Level

Some congenital conditions have shown a declining trend across Europe. Table 10 outlines congenital anomalies for which a declining prevalence has been reported and discusses possible explanations for these observed trends.

Therefore, some of these trends are considered to be attributable to improved prenatal care, diagnostic accuracy or classification practices. However, further investigation is necessary to fully understand these trends. A brief overview of each condition and its relevance to TToP is discussed below.

Hydrocephaly—Hydrocephaly involves an accumulation of cerebrospinal fluid in the brain. This condition can lead to an increase in intracranial pressure and potential damage to brain tissue. Mild cases of hydrocephaly can often be managed with shunting, but more severe forms may lead to developmental delays, seizures and poor long-term prognosis. In cases where hydrocephaly is diagnosed in conjunction with other severe malformations (e.g., agenesis of the corpus callosum, severe developmental delay), the severity of the condition may lead the mother to take into consideration the therapeutic abortion as a valid solution [194,195]. Early detection allows for a decision to be made sooner in the pregnancy in order to prevent potential long-term suffering for the child, but also for the family.

Severe Microcephaly—Severe Microcephaly is defined as a head circumference more than 3 standard deviations below the mean for sex and gestational age. It is often associated with intellectual disability as well as other neurological deficits. This condition can be extremely debilitating (developmental delays, seizures and neurological impairments). Some children may survive and reach adulthood, but their quality of life is usually greatly affected [196]. In cases where microcephaly is diagnosed early in pregnancy (especially when associated with other neurological malformations), the severity of the condition may lead the mother to seek therapeutic abortion. A poor prognosis, as well as no viable treatment options to improve quality of life, represent factors that may lead the mother to choose TToP.

Anophthalmos—Anophthalmos is the congenital absence of one or both eyes, resulting in blindness. This birth defect does not necessarily affect life expectancy, but it may be associated with other serious anomalies (such as brain malformations or developmental delays). Therapeutic abortion may be considered in cases where anophthalmos is part of a broader syndrome with a poor prognosis or severe developmental delay. However, the mother may prefer to continue the pregnancy if the fetus has isolated anophthalmos (particularly if diagnosed early in pregnancy), as long-term management options such as prosthetic eyes or early interventions may improve the quality of life [197].

Congenital glaucoma—This condition is characterized by elevated intraocular pressure and is usually diagnosed in infancy. It is well known that congenital glaucoma can lead to blindness. In severe cases where blindness occurs (especially when associated with other syndromes), therapeutic abortion may be considered. On the other hand, if interventions (e.g., surgery or medication to control intraocular pressure) are performed early, many children with isolated congenital glaucoma can have functional vision [198].

Atrial septal defect (ASD)—Atrial septal defect is characterized by a hole in the wall that separates the two atria of the heart, allowing abnormal blood flow between the heart’s chambers. Most cases of ASD are asymptomatic or can be successfully managed with surgery in childhood, with excellent long-term outcomes. Isolated ASD is not in itself a standard indication for therapeutic termination of pregnancy. However, it may enter counseling discussions when the defect is large, deemed unsuitable for effective surgical repair, or when it occurs in association with severe syndromic or multisystem disease that significantly worsens the overall prognosis [199].

Tricuspid atresia and stenosis—Tricuspid atresia and stenosis are congenital heart defects that affect the tricuspid valve and right ventricle [200]. Therapeutic abortion might be considered in severe cases where surgical repair is not possible, due to the high risk of death early in life [201]. However, with advanced surgical techniques and early intervention, many children with these conditions can survive and can live relatively normal lives.

Cleft palate—Cleft palate is a defect in the soft and/or hard palate that can cause difficulty with feeding, speech and hearing and typically requires one or more surgeries for correction [202]. In the vast majority of cases, cleft palate can be successfully repaired with surgery, and children go on to live normal lives. Accordingly, isolated cleft palate is not in itself a typical indication for therapeutic termination of pregnancy. However, it may enter counseling discussions when it occurs as part of a severe syndrome [203] or when the malformation leads to life-threatening conditions (e.g., severe airway obstruction refractory to postnatal intervention) or when associated with major neurological or skeletal impairment.

Gastroschisis—Gastroschisis involves the protrusion of abdominal contents through a defect in the abdominal wall. It is typically seen in the offspring of younger mothers. This condition is usually treatable through surgical intervention shortly after birth, with good survival in isolated cases, but in some cases, it can be associated with other serious anomalies or prematurity that might complicate recovery [203]. In this context, gastroschisis is not in itself a standard indication for therapeutic termination of pregnancy, but it may enter counseling discussions when associated with severe syndromic or multisystem disease that significantly worsens the overall prognosis.

Hypospadias—Hypospadias is a condition in boys where the urethral opening is abnormally located on the underside of the penis. This condition often requires surgery for correction [204]. Most cases have a favorable long-term outcome and do not lead to life-threatening complications. Accordingly, hypospadias on its own is not a typical indication for therapeutic termination of pregnancy, but it may enter counseling discussions when it forms part of a broader syndrome or multisystem disorder with major implications for survival or long-term quality of life.

Pierre Robin sequence—Pierre Robin sequence is characterized by micrognathia, glossoptosis and airway obstruction. It is often associated with a posterior cleft palate. Most children with Pierre Robin sequence can be managed with timely airway and feeding interventions, and the cleft palate can usually be surgically corrected later in childhood, so isolated cases generally have a heterogeneous but often compatible prognosis. From the perspective of therapeutic termination of pregnancy, these anomalies may enter counseling discussions mainly when there is severe airway compromise anticipated despite optimal postnatal care, or when they occur in the context of a severe syndromic or multisystem condition (for example, with major neurological and/or skeletal malformations) that leads to a clearly poor prognosis [205].

Skeletal dysplasias—Skeletal dysplasias are genetic disorders characterized by abnormal bone development. Some forms of skeletal dysplasia can lead to severe deformities or reduced life expectancy [206]. Therefore, therapeutic abortion may be considered in extreme cases (where the prognosis is poor). On the other hand, less severe forms of skeletal dysplasias often allow for a normal lifespan.

## 9. Ethical, Religious and Legal Considerations of TToP/VToP

Induced abortion is influenced by medical, ethical, religious, social, economic, as well as legal factors. People’s opinions on abortion often fall into 2 main perspectives: the fetus’s right to life versus the woman’s right to bodily autonomy. While legal and safe abortions carry minimal health risks, global estimates for 2010–2014 indicate that 45% of induced abortions were unsafe. This percentage corresponds to approximately 25 million unsafe abortions per year [207,208].

Abortion raises ethical questions regarding a woman’s right to her own body versus the fetus’s right to life. In light of these, Judith Jarvis Thomson has argued that a woman should not be forced to carry an unwanted pregnancy, using her famous violinist analogy to emphasize the autonomy of the woman [209]. Furthermore, she discusses the distinction between a fetus and a human being, pointing out that for those who support abortion, the foetus is not considered a living person until a certain developmental stage [209]. Carol Gilligan, on the other hand, highlights the dilemma of choice women face when deciding to undergo an abortion, emphasizing the conflict between the woman’s self-interest and the potential harm to others. Through interviews with 29 women from diverse backgrounds, Gilligan found that the central moral issue revolves around the tension between the woman’s self-interest and the possible consequences for those around her [210,211].

Religious perspectives generally oppose abortion and consider this act as morally and spiritually unacceptable [212]. Christianity views abortion as homicide, equating it with attacking Jesus Christ and God (as it is seen as a denial of faith). Islam prohibits abortion based on the belief in the sanctity of life (as outlined in the Koran) (Chapter 6, Verse 151). Buddhism considers abortion a negative act, but it allows it for medical reasons. Judaism condemns abortion and considers it a mortal sin. Hinduism views it as both a crime and the greatest sin.

Legal approaches to abortion vary worldwide, reflecting differing cultural and political attitudes. In 66 countries (25.5% of the global population), abortion is prohibited except when the mother’s life is at risk. A less restrictive framework is seen in 59 countries (13.8%), where abortion is allowed only for health reasons (broadly defined by the World Health Organization). In 13 countries (21.3%), abortion is permitted on socio-economic grounds (such as cases of rape, incest or financial hardship). Meanwhile, 61 countries (39.5%) have an unrestricted approach, allowing abortion upon request within a specified period, usually up to 12 weeks [2].

In Romania, abortion is regulated by Article 201 of the Penal Code. Voluntary termination of pregnancy is legal up to 14 weeks of gestation, but it has to be performed by a licensed obstetrics–gynecology specialist within authorized medical facilities. Beyond 14 weeks, abortion is permitted only for therapeutic reasons, such as safeguarding the health or life of the pregnant woman or addressing severe fetal anomalies. In these cases, the procedure must be carried out by a qualified specialist, and there is no explicit gestational limit for therapeutic abortions, though clinical guidelines typically consider up to 24 weeks. Importantly, Romanian law does not criminalize the pregnant woman for undergoing an abortion, regardless of the circumstances. However, an abortion that is performed without the woman’s consent or outside the legal framework can result in criminal penalties for the provider (such as imprisonment and revocation of medical practice rights) [213].

Restrictive laws do not prevent abortions, but drive them underground. As a result, this leads to increased health risks. A large percentage of unsafe abortions occur in developing countries. Consequently, access to safe abortion remains a key issue in reproductive health and human rights discussions [208].

## 10. Conclusions

The therapeutic termination of pregnancy (TToP) represents a crucial element of modern reproductive healthcare because it provides a medically supervised choice when maternal health is endangered or when severe fetal abnormalities are detected. The availability of advanced prenatal screening and diagnostic tools including serum markers, ultrasound, cell-free DNA testing, chorionic villus sampling and amniocentesis has greatly improved the ability to detect conditions early, supporting better decision-making. The methods enable doctors to determine risks with high accuracy and perform pregnancy terminations (medical or aspiration/surgical abortions) at safer times. However, the decision to pursue TToP is rarely based on medical findings alone. First-trimester combined screening (nuchal translucency measurement together with serum markers such as PAPP-A and free beta-hCG) provides early risk stratification, while cell-free fetal DNA testing offers high sensitivity for common aneuploidies (trisomy 21, 18 and 13). Invasive procedures, such as chorionic villus sampling and amniocentesis, remain the gold standard for definitive chromosomal diagnosis. Preimplantation genetic diagnosis further extends these options for couples undergoing in vitro fertilization. Genetic counseling plays a central role in this process, as it ensures that patients receive detailed information about the risks, benefits and limitations of each test, thereby supporting truly informed reproductive decisions.

The rates of abortion and access to abortion services vary considerably based on sociodemographic factors such as age, marital status, educational attainment, employment and urban versus rural residence. Cultural stigmas, limited access to contraception and legal restrictions, are factors that increase the incidence of unsafe procedures. Younger women, students and individuals who do not have an adequate financial or family support experience heightened vulnerability. Immigrant women and ethnic minorities face disproportionately higher abortion rates due to economic hardship and systemic barriers to healthcare access. Intimate partner violence and gender-based violence further contribute to unwanted pregnancies and repeated abortions. Religious beliefs also play a dual role: while strong religious affiliations may reduce the likelihood of seeking abortion, they can also intensify feelings of guilt and psychological distress in women who do undergo the procedure. Women with pre-existing psychiatric conditions (such as anxiety, affective disorders, psychotic or trauma-related disorders) have higher rates of therapeutic abortion. Notably, the majority of mental health problems appear before the procedure rather than developing as a consequence of it. Women with borderline personality disorder, substance use disorders, eating disorders and childhood psychiatric disorders (such as ADHD and conduct disorder) are at particular risk of unplanned pregnancies and subsequent termination. The procedure leads to emotional relief for most patients, but some women develop lasting symptoms which include depression, anxiety, PTSD and substance misuse. Second-trimester TToP carries a greater psychological burden compared to first-trimester procedures, largely due to stronger maternal-fetal attachment and the surgical nature of the intervention. Risk factors for adverse psychological outcomes include coercion, pre-existing psychopathology, limited social support, strong maternal–fetal attachment and the absence of contraceptive use prior to pregnancy. Emotional and social support is a major protective factor. On the other hand, its absence can intensify feelings of guilt, grief and psychological distress.

The legal, ethical and religious frameworks regarding TToP vary widely across countries, but consistently shape access and perception. The ethical debate fundamentally revolves around the tension between the fetus’s right to life and the woman’s right to bodily autonomy. While 61 countries (representing 39.5% of the global population) currently allow abortion upon request within a specified period, 66 countries prohibit it except when the mother’s life is at risk. In Romania, voluntary termination of pregnancy is legal up to 14 weeks of gestation, and therapeutic abortion beyond this limit is permitted only for medical reasons with no explicit gestational ceiling, though clinical guidelines typically consider up to 24 weeks. Laws that restrict abortion do not lower the number of abortions, but rather force women to seek dangerous procedures that increase their health risk. According to the World Health Organization, an estimated 45% of all induced abortions worldwide are unsafe, corresponding to approximately 25 million unsafe procedures per year. Unsafe abortions, predominantly occurring in developing countries, remain a major and preventable contributor to maternal morbidity and mortality.

Between 2013 and 2022, EUROCAT reported increasing trends for conditions such as Double Outlet Right Ventricle (DORV), Caudal Regression Sequence (CRS), Trisomy 13 and Trisomy 18, which represent severe anomalies that may lead to therapeutic termination of pregnancy. The increase in DORV cases is considered to be due to improved coding, while CRS has been linked to higher rates of maternal diabetes and obesity. The increasing trends for Trisomy 13 and 18 are associated with a better access to non-invasive prenatal testing. In contrast, conditions like hydrocephaly, severe microcephaly, anophthalmos, congenital glaucoma and atrial septal defect showed decreasing trends. This finding may be attributed to improvements in terms of prenatal care, better classification or earlier detection (which in turn may influence TToP decisions in severe cases). Conversely, decreasing trends were observed for conditions such as gastroschisis, hypospadias, cleft palate, Pierre Robin sequence, skeletal dysplasias, tricuspid atresia and stenosis, congenital glaucoma and atrial septal defect. These declines are likely attributable to improvements in prenatal care, more accurate classification systems and earlier detection, although the precise mechanisms underlying some of these trends warrant further investigation. It should be noted that many of the conditions with decreasing prevalence (such as isolated cleft palate, hypospadias, gastroschisis and atrial septal defect) are not in themselves standard indications for TToP, but may enter counseling discussions when they occur in the context of severe syndromic or multisystem disease.

The management of TToP should not be limited to clinical practice, but should also include psychological support, ethical reflection and social awareness. Peri-procedural emotional support is essential, particularly in second-trimester TToP, and should involve trained staff, respectful communication and ensured privacy to reduce acute distress. Post-procedural follow-up should include systematic psychological evaluation, especially in patients with identified risk factors (e.g., pre-existing psychiatric disorders, limited social support or coercion). Grief counseling and mental health referral are recommended as integral components of standard care to reduce long-term psychological morbidity. Contraceptive counseling is necessary in order to prevent repeated abortion procedures. Mental health services are recommended to ensure the long-term well-being of the mother.

In conclusion, TToP is more than just a medical act; it is a complex, deeply human decision. The findings of this review reinforce the importance of a multidisciplinary, evi-dence-informed approach that integrates clinical expertise, psychological support, ethical reflection and legal awareness. Ensuring safe, equitable and compassionate access to TToP, regardless of socio-economic background, geographic location or cultural context, remains a critical component of reproductive healthcare. Future research should focus on standardizing psychological screening protocols for women undergoing TToP, evaluating the long-term mental health outcomes through prospective longitudinal studies and addressing the persistent disparities in access to safe abortion services across different regions and populations.

## Figures and Tables

**Table 1:** Major fetal structural anomalies detectable during 11–13 + 6 weeks ultrasound scan and their prognostic implications.

System/Region	Fetal Anomaly	Ultrasound Marker/Findings	Detection Feasibility at 11–13 + 6 Weeks	Prognostic Implication	References
**Central Nervous System**	Acrania/Anencephaly	Absence of calvarium and exposed brain tissue	Clearly detectable from 11 weeks	Lethal	[19]
	Holoprosencephaly	Fused thalami, single ventricle	Severe forms identifiable from 11–12 weeks onward	Severe neurological impairment or death	[20]
	Dandy–Walker malformation	Enlarged posterior fossa, absent cerebellar vermis	May be suspected near 13 weeks; full dx. often later	Hydrocephalus, motor delay	[21]
	Spina bifida (open)	Lemon sign, banana sign, spinal defect	Early cranial markers visible ~13 weeks	Variable neurologic disability	[22,23]
	Encephalocele	Extracranial brain herniation through skull defect	May be detected after 11–12 weeks	Often severe; poor prognosis if large or syndromic	[24]
	Ventriculomegaly/Hydrocephalus	Enlarged lateral ventricles	Incipient signs possible; definitive dx. often later	Variable; may progress or normalize	[25,26]
**Cardiovascular System**	Atrioventricular septal defect (AVSD)	Abnormal 4-chamber view, reversed DV or tricuspid regurgitation	Suspicion possible at 11–13 + 6 weeks	Often syndromic; possible heart failure	[27,28]
	Cardiomegaly	Globally enlarged cardiac silhouette	May be seen if associated with hydrops	Suggests fetal compromise, anemia, infection	[29]
	Pericardial effusion	Anechoic rim around heart	Early marker of hydrops or infection	Often poor prognosis in context	[30]
	Tricuspid regurgitation	Abnormal flow across tricuspid valve	Detectable via Doppler	Strong marker for trisomy 21	[31]
	Reversed ductus venosus flow	Reversed A-wave on DV Doppler	Technically feasible with skilled scanning	Associated with aneuploidy and heart defects	[32,33]
	Bradycardia/Tachycardia	Heart rate < 110 beats per minute (bpm) or >160 bpm sustained on M-mode or Doppler	Readily detectable; may be transient or persistent	Isolated findings often benign; persistent forms may suggest genetic or cardiac disease	[34,35]
**Craniofacial Region**	Facial cleft	Disruption of upper lip contour, possible palate defect	Bilateral clefts may be visible near 13 weeks	Often surgically correctable if isolated	[36]
	Absent nasal bone	Non-visualization of echogenic nasal bone	Readily detectable at 12–13 weeks	Marker for trisomy 21, 18	[37]
**Thoracoabdominal Region**	Congenital diaphragmatic hernia	Stomach in chest, mediastinal shift	Suspicion possible ~13 weeks	Severe pulmonary hypoplasia	[38,39]
	Body stalk anomaly	Thoracoabdominal wall defect, absent cord	Easily detected	Uniformly lethal	[40]
	Omphalocele	Midline herniation, membrane-covered, liver possibly included	Clearly visible; consider liver inclusion for prognosis	Often syndromic	[41]
	Gastroschisis	Bowel loops outside abdomen, no membrane	Readily identifiable	Isolated form has good prognosis	[42]
	Megacystis	Bladder ≥ 7 mm on sagittal view	Easily measured	May resolve or progress to obstruction	[43]
	Hydrops fetalis	Skin edema, ascites, effusions	Clearly visible; may evolve rapidly	Often fatal; multiple etiologies	[44]
**Musculoskeletal System**	Lethal skeletal dysplasia	Short limbs, narrow thorax, abnormal bone ossification	May be detected >13 weeks	Lethal in many cases	[45,46]
	Limb abnormalities (e.g., amelia)	Absent or severely shortened limbs	Identifiable from 12 weeks	Syndromic or isolated	[47]
	Amniotic band syndrome	Limb entrapment, amputations, constriction rings	Visible with major defects; subtle forms harder to detect	Variable severity	[48]
**Other/Markers**	Increased nuchal translucency (isolated)	NT ≥ 95th percentile or >3.5 mm	Routinely measured; standardized technique	Marker for chromosomal anomalies, heart defects	[49,50]
	Umbilical cord anomalies	Single umbilical artery, abnormal insertion	Possible from 13 weeks with Doppler	May indicate syndromes or intrauterine growth restriction	[51]

**Table 2:** Major fetal anomalies detectable with ultrasound screening in the second trimester of pregnancy and their prognostic implications.

System/Region	Fetal Anomaly	Ultrasound Findings	Detection Window	Prognostic Implication	References
**Central Nervous System**	Spina bifida (open)	Posterior vertebral defect, lemon sign, banana sign	18–20 weeks	Variable disability, hydrocephalus, motor impairment	[23,60]
	Hydrocephalus	Enlarged lateral ventricles (>10 mm)	18+ weeks	May progress; associated with genetic	[26,61]
	Agenesis of corpus callosum	Absent cavum septi pellucidi, colpocephaly	20–22 weeks	May be isolated or part of syndrome	[62]
	Holoprosencephaly	Single midline ventricle, absent interhemispheric fissure	18+ weeks	Often severe/profound neurologic impairment	[63]
	Dandy–Walker spectrum	Enlarged cisterna magna, vermian hypoplasia	20–22 weeks	Developmental delay, ataxia	[64]
	Encephalocele	Herniation of brain tissue through skull defect	18+ weeks	Prognosis depends on size and content	[24,65]
**Cardiovascular System**	Hypoplastic left heart syndrome (HLHS)	Small/absent left ventricle, reversed flow in aortic arch	18–22 weeks	Lethal without surgery; major neonatal interventions	[66]
	Tetralogy of Fallot	Overriding aorta, VSD, pulmonary stenosis	18–22 weeks	Variable outcomes; often repairable	[67,68]
	Atrioventricular septal defect (AVSD)	Common atrioventricular valve, absent septa	18–22 weeks	Associated with Down syndrome; surgical repair possible	[69]
	Ebstein anomaly	Apical displacement of tricuspid valve	18–22 weeks	May cause fetal hydrops or heart failure	[70]
**Craniofacial Region**	Cleft lip ± palate	Discontinuity of upper lip and/or palate	18–22 weeks	May be isolated or syndromic; surgical correction possible	[71]
	Micrognathia	Small chin, abnormal jaw angle	18–22 weeks	Often marker for syndromes (e.g., Pierre Robin, trisomies)	[72]
	Cyclopia/proboscis	Single orbit, absent nose	18–22 weeks	Lethal (alobar holoprosencephaly)	[73]
**Thoracoabdominal Region**	Congenital diaphragmatic hernia (CDH)	Abdominal contents in chest, lung hypoplasia	18–22 weeks	Pulmonary hypoplasia, high neonatal mortality	[74]
	Omphalocele	Herniated liver/intestines with membrane	18–22 weeks	Syndromic in many cases (e.g., trisomy 13/18)	[75]
	Gastroschisis	Free bowel loops without membrane	18–22 weeks	Typically isolated; surgical correction possible	[42]
	Body stalk anomaly	No cord, severe wall defect, fetus adherent to placenta	18+ weeks	Uniformly lethal	[76]
**Renal/Urogenital System**	Bilateral renal agenesis	Absent kidneys, anhydramnios, empty bladder	20–22 weeks	Lethal (Potter sequence)	[77]
	Polycystic kidneys (ARPKD)	Bilaterally enlarged echogenic kidneys	20–22 weeks	Lethal if severe; may lead to anhydramnios	[78]
	Obstructive uropathy (severe)	Keyhole sign, megacystis, hydronephrosis	18–22 weeks	Can lead to renal failure, pulmonary hypoplasia	[79]
**Musculoskeletal System**	Thanatophoric dysplasia	Very short limbs, bowed femora, narrow thorax	18–22 weeks	Lethal skeletal dysplasia	[80]
	Achondrogenesis	Short limbs, poor ossification	18–22 weeks	Lethal skeletal dysplasia	[81,82]
	Clubfoot (talipes)	Persistent foot deviation	18–22 weeks	May be isolated or syndromic	[83]
	Limb reduction defects	Absence or shortening of limbs	18–22 weeks	May be isolated or syndromic	[84]
**Others/Syndromes**	Hydrops fetalis	Skin edema, ascites, pleural/pericardial effusion	18–22 weeks	Indicates cardiac, genetic or infectious pathology	[44]
	Fetal tumors (e.g., sacrococcygeal teratoma)	Solid/cystic mass near spine or sacrum	18–22 weeks	May cause hydrops or dystocia depending on size	[85]
	Conjoined twins	Fused thoraxes/abdomens, shared organs	18–22 weeks	Lethal or surgically inoperable in most cases	[86,87]

**Table 3:** Sociodemographic Determinants of Abortions.

Factor	The Association with Abortions	References
**Age**	Women between the ages of 20 and 34 show the highest rates of TToP/VToP (corresponding to peak fertility). On the other hand, women under 20 years of age, as well as those over 34 years of age, are more likely to undergo abortion due to factors such as lower fertility and an increased likelihood of genetic conditions. The majority of abortions in developing countries occur among women under 34. Various factors, such as delayed recognition, as well as socio-economic and legal barriers, might cause adolescents to seek abortion later. Moreover, a restricted access to medical services can lead to a higher risk of unsafe abortions, especially among adolescents.	[96,97,98,99,100,101]
**Marital Status**	In western countries, divorced women have three times higher abortion rates than unmarried women and five times higher rates than married or widowed women. The absence of a partner or supportive family significantly influences abortion decisions. The majority of abortions in developing countries occur among married (46.4%) or single (41.6%) women due to cultural norms that discourage divorce and single parenthood, despite legal acceptance. Additionally, an unstable relationship, as well as a lack of financial or emotional support from a partner, contributes significantly to abortion rates.	[99,100,102,103]
**Education Level**	It has been observed that, in some cases, highly educated women delay childbearing, which leads to a higher need for termination of pregnancy (due to age-related fertility issues). In contrast, lower-educated women tend to seek abortions due to financial insecurity and lack of access to contraception.	[97,101,103,104]
**Employment & Income**	Women from western countries who prioritize their career have higher abortion rates. On the other hand, the majority of abortions in developing countries occur among housewives and students. Low-income women are at higher risk, with economic hardship being a primary reason for termination. The abortion rates among women who earn less than the federal poverty level exceed those of wealthier women by five to six times. In developing countries, the highest risk for termination is among women with unstable jobs.	[97,99,100,101,103,105,106,107]
**Number of Children**	Women with two or more children are significantly more likely to seek TToP/VToP (often due to financial constraints or difficulties managing additional offspring). It has been observed that family planning plays a crucial role in abortion decisions. Many women who seek abortion have not accessed contraception services within the previous two years. Unplanned pregnancies that lead to abortion among adolescents are correlated with inadequate sexual education and limited access to birth control methods.	[99,100,105]
**Urbanization**	Higher termination of pregnancy rates in urban areas are linked to better access to healthcare, legal abortion services and a more pronounced anti-natal mindset. Urban living often increases the perceived financial burden of raising children (which leads to higher abortion rates). Moreover, it is well known that government family planning programs and media exposure in cities also contribute to abortion decisions. In contrast, the cultural preference for bigger families in rural areas leads to higher birth rates compared to urban areas.	[97,100,104]
**Immigration & Ethnicity**	Immigrant women and ethnic minorities have higher abortion rates due to economic hardship and barriers to healthcare. In some countries, migrant women have twice the abortion rate of native-born women, even when adjusted for income. Ethnic minorities (particularly in the U.S.) have a higher likelihood of undergoing abortion at a later stage of pregnancy due to a broad range of factors (systemic inequalities in healthcare access, contraceptive availability and socio-economic disparities).	[103,107,108,109]
**Religious Beliefs**	Strong religious affiliations reduce abortion access, while lower religiosity increases the likelihood of seeking termination of pregnancy. Some religious communities provide psychological support for women who choose to carry their fetuses to term, especially when the fetus has an anomaly. However, women who have strong religious beliefs may feel intense guilt along with psychological distress after getting an abortion.	[108,110,111]
**Intimate Partner Violence (IPV) & Gender-Based Violence**	The experience of IPV and sexual violence is considered to be an important factor for abortion. Women who suffer from gender-based violence through forced sex and coercion have a higher likelihood of unwanted pregnancies and repeated abortions. In some cases, termination of pregnancy serves as a means of escaping an abusive relationship. Clinicians should assess these factors when evaluating patients, as IPV is also linked to adverse birth outcomes and maternal mental health issues.	[104,112]

**Table 4:** Psychiatric Conditions Associated with Increased Likelihood of TToP.

Psychiatric Condition	Relevance to TToP and Reproductive Risk	References
**Anxiety Disorders**	Panic disorder, social phobia, generalized anxiety disorder and tocophobia are common. The fear of pregnancy or childbirth may arise from cultural influences, intergenerational transmission, or past trauma (such as childhood sexual abuse); thus, some women may avoid seeking obstetric care.	[117,118,119]
**Affective Disorders**	Feelings of inadequacy regarding motherhood, previous suicidal ideation or attempts, and concerns about medication effects on fetal development (e.g., fluoxetine, sodium valproate). Fear of teratogenic risks may lead to higher TToP rates among medicated women, despite the possibility of alternative treatments and antenatal monitoring.	[117,118,120,121]
**Borderline Personality Disorders (BPD)**	Impulsive behaviors, unstable relationships and impaired decision-making contribute to early and unprotected sexual activity. Furthermore, high rates of substance abuse, sexual trauma and STDs increase reproductive risks and the probability of abortion. Factors influencing pregnancy outcomes include socio-economic disadvantage, young maternal age and involvement in high risk environments (such as sex work).	[122,123]
**Post-Traumatic Stress Disorder (PTSD)**	The decision to terminate pregnancy may be influenced by PTSD, especially in cases of rape, coercion or unstable relationships. Women with PTSD also experience an increased risk of post-abortion distress, which is even higher if they have prior psychiatric vulnerabilities. Some women may also deal with reproductive coercion, such as being forced into pregnancy or having their contraception sabotaged, which may complicate their reproductive decisions.	[117,124,125]
**Psychotic Disorders**	Limited knowledge and access to contraception, increased sexual activity due to deinstitutionalization and second-generation antipsychotics, and lower likelihood of stable relationships contribute to higher TToP rates. Socio-economic disadvantages, younger age at conception and the absence of social support are known as common factors that may influence abortion decisions. Women with schizophrenia also face higher risks of pregnancy-related complications, which may include maternal health issues and fetal abnormalities (possibly linked to genetic predispositions or antipsychotic medication effects). Addressing reproductive health education and contraceptive access is crucial in this population.	[126,127,128,129]
**Substance Use Disorders (SUDs)**	Higher levels of sexual risk-taking, multiple sexual partners and an inconsistent way of using contraception increase TToP risk, particularly among injecting drug users with health complications (HIV, hepatitis C). Concerns about fetal harm from substance exposure (e.g., preterm birth, anomalies) may also influence decisions of these patients. Binge drinking and diagnosed addiction significantly increase abortion likelihood.	[118,130,131]
**Childhood Psychiatric Disorders (e.g., ADHD)**	Childhood impulse-control disorders (e.g., ADHD, conduct disorder, oppositional defiant disorder) significantly increase the risk of induced abortion. An early onset of psychiatric conditions is associated with poor school performance and low education, as well as high rates of unprotected sex and STDs. Adolescents with conduct disorders are also at higher risk of sexual violence. Targeted mental health interventions and comprehensive sex education could reduce reproductive health risks.	[117,118,132]
**Eating Disorders (e.g., anorexia, bulimia)**	Women with eating disorders such as anorexia nervosa (AN) and bulimia nervosa (BN) have a higher likelihood of unwanted pregnancies and abortion, partly due to menstrual irregularities (which can lead to a false sense of security regarding contraceptive use). These women are at increased risk of gynecological issues like infertility, pregnancy complications (e.g., preterm birth, low birth weight) and gestational diabetes. Studies show that women with AN and BN have higher rates of teenage pregnancies, unplanned pregnancies, as well as repeat abortions. Moreover, their eating disorders may impair their ability to recognize the need for contraception, especially if they experience oligomenorrhea or amenorrhea. Poor sexual education is a contributing factor; women with eating disorders often have insufficient awareness of the risk of pregnancy during menstrual irregularities. Increased risks of miscarriage and abortion are reported, especially in women with a later onset of eating disorders (18–22 years old).	[133,134]
**Paraphilic or Compulsive Sexual Behaviors**	Women with compulsive sexual behaviors or paraphilias often experience dysfunctional sexual relationships, leading to a higher risk of unwanted pregnancies. These behaviors like sexual submission or victimization can make women unable to develop healthy and affectionate relationships, and they are therefore likely to have unprotected sex. Women with such problems are at a higher risk of sexually transmitted infections, infertility, and obstetric complications like miscarriage and ectopic pregnancies, which are sometimes a result of sexual trauma. Consequently, many seek abortion due to the psychological distress and health concerns associated with these conditions.	[135]

**Table 5:** Psychological Outcomes Following TToP.

Psychiatric Outcome	Post-TToP Impact and Predictive Factors	References
**Depression & Anxiety**	Most women experience mood improvement post-TToP, but ~10% develop persistent depression or anxiety. Long-term psychological distress is more common in patients with poor partner support, relational conflicts, psychiatric history, young age, multiparity or poor social networks. Women from cultures or religious groups opposing abortion may also be at higher risk. Many cases of post-TToP depression are due to pre-existing mood disorders rather than the procedure itself. Early identification and management of socio-cultural and psychological risk factors during pregnancy are recommended in order to prevent adverse mental health outcomes.	[105,139]
**Mania & Bipolar Disorder**	Abortion may trigger manic episodes in women with bipolar disorder, but evidence is inconclusive. Isolated reports have described the potential risk of converting bipolar II disorder to bipolar I following abortion. On the other hand, no significant increase in hospitalization rates post-TToP was found in broader studies. Therefore, the impact on the disease progression may be minimal for most patients.	[114,147]
**Post-Abortive Psychosis**	Rare but documented phenomenon. The hospitalization rate for post-TToP psychosis is 0.3/1000, significantly lower than the 1.7/1000 rate for postpartum psychosis. The onset is usually within 2 weeks after the abortion and is similar to puerperal psychosis. Cases have been observed in women with schizophrenia, but also in those with no history of psychosis. Hormonal fluctuations (particularly a rapid drop in estradiol levels) are believed to contribute to the onset of psychosis. Individual vulnerability, genetic predisposition and psychological distress may also be risk factors.	[146]
**PTSD & Post-Traumatic Symptoms**	TToP can lead to post-traumatic stress symptoms (PTSS) or PTSD. While some cases resolve within six months, others might persist for years. Approximately 64.5% experience PTSS two weeks post-TToP, but only 22% develop long-term PTSD. Individual vulnerability, pre-existing psychiatric conditions as well as the circumstances of the abortion influence the risk of PTSD.	[143,144]
**Substance Use Disorders (SUDs)**	SUDs were correlated with a higher risk of alcohol, tobacco and drug abuse post-TToP. Possible causes include emotional distress, pre-existing mental health conditions, social stigma and lack of support. However, causality remains debated and is not well understood, as substance use may predate abortion in some cases.	[145]

**Table 6:** Risk Factors for Negative Psychological Outcomes after TToP.

Determinant	Psychological Impact Post-TToP	References
**Medical/genetic indications**	The psychological distress faced by women who opt to end pregnancies due to fetal anomalies or maternal health risks exceeds the distress experienced by those undergoing VToP for social or ethical reasons. These cases involve complex issues along with feelings of grief and moral conflicts which produce elevated depression, anxiety and post-traumatic symptoms. Adequate counseling and psychological support are recommended to help minimize adverse outcomes for these patients.	[148,149]
**Demographics and socio-cultural background**	Single, nulliparous, immigrant or minority women under 30 (particularly adolescents) face a higher risk of developing negative psychological outcomes. Young women often face intense emotional struggles. Furthermore, cultural and religious factors may amplify feelings of guilt and regret. It is considered that strong religious beliefs do not necessarily increase guilt, but negative cultural attitudes toward abortion can heighten psychological distress.	[150,151]
**Coercion**	Women coerced into abortion by partners or family members have a higher risk of developing negative psychological effects (such as depression, guilt and PTSD). On the other hand, those who make an independent decision tend to have better mental health outcomes.	[151,152]
**Pre-existing psychopathology**	Women who experience psychological distress before an abortion combined with low self-esteem and high alienation levels become more susceptible to negative mental health consequences. It was also reported that women with strong resilience show better ability to adapt positively following a TToP procedure.	[153,154]
**Emotional support**	Perceived emotional support improves psychological recovery, whereas partner absence during abortion has been linked to increased depression and guilt. Weak family bonds, work stress and partner abandonment further exacerbate negative psychological effects. A lack of social support can also contribute to feelings of isolation and regret.	[142,151]
**Maternal attachment to the fetus**	The intensity of maternal thoughts about the baby correlates with psychological distress post-TToP. It is known that women who establish strong emotional bonds with their fetuses before abortion procedure face a higher risk of experiencing negative feelings, such as grief, guilt and depressive symptoms.	[151]
**Illegal abortion**	Women undergoing illegal abortions report higher rates of guilt, depressive symptoms and post-traumatic distress. These symptoms might also be intensified by the lack of medical oversight, as well as secrecy and fear of legal repercussions.	[155]
**Lack of contraception**	Women who did not use contraception before pregnancy often experience significant guilt and depression post-TToP. Therefore, post-abortion contraceptive education plays a crucial role in stopping repeat abortions and decreasing feelings of self-blame.	[151]

**Table 7:** Common complications of medical abortion and recommended clinical management.

Complication	Management
**Heavy bleeding and/or severe cramping**	These are common side effects but may require intervention when excessive or prolonged. Management includes repeat misoprostol dosing, administration of NSAIDs for pain, and uterine aspiration if bleeding is uncontrolled. Furthermore, blood transfusion may be required in rare cases of significant hemodynamic compromise [169].
**Failure of medical abortion**	This may be suspected in cases with a persistence of gestational tissue or ongoing pregnancy symptoms. Treatment options include a second course of misoprostol or surgical uterine aspiration (depending on clinical factors and patient preference) [170].
**Infection (e.g., endometritis)**	May present with fever persisting beyond 24 h post-misoprostol, abdominal or pelvic pain, vaginal discharge as well as uterine (or adnexal) tenderness. Management involves uterine aspiration if retained tissue is suspected, and administration of antibiotics as per Centers for Disease Control and Prevention (CDC) pelvic inflammatory disease (PID) protocols. In cases of systemic involvement and/or hemodynamic instability, hospital admission and aggressive antibiotic therapy are required [165].
**Ectopic pregnancy**	Although rare, it must always be ruled out when expected post-abortion symptoms do not occur, or when no products of conception are passed. The management involves immediate referral and treatment—typically with methotrexate for stable patients or surgical intervention for unstable cases [165].

**Table 8:** Common complications of aspiration abortion and recommended clinical management.

Complication	Management
**Heavy bleeding**	May be managed with NSAIDs, repeat misoprostol administration, uterine aspiration as well as blood transfusion when necessary [165,169]. A structured clinical approach using the “6 Ts” can guide management: - **Tone**: Uterine massage and administration of uterotonics (e.g., methergine, misoprostol). - **Tissue**: The confirmation of complete evacuation of uterine contents is necessary. - **Trauma**: Any cervical or vaginal tears must be evaluated and repaired. - **Thrombin**: Review coagulation status. Perform relevant blood tests (e.g., CBC, INR). - **Treatment**: Use IV fluid bolus and intrauterine tamponade with a Foley catheter balloon. - **Transfer**: If the patient remains unstable, arrange urgent transfer to a higher level of care [174].
**Incomplete abortion**	Can be managed with additional doses of misoprostol or repeat aspiration, particularly in cases with persistent bleeding, pain, or suspected infection [171,174].
**Infection (endometritis)**	Such cases can be treated with antibiotics (according to CDC pelvic inflammatory disease guidelines). Uterine aspiration may be necessary if retained tissue is suspected. Testing for gonorrhea and chlamydia is also recommended. Ultrasound may aid in diagnosis and guide further management [175].
**Ectopic pregnancy**	Should be suspected when no products of conception are retrieved. Diagnosis may require serial hCG levels or ultrasound. Management includes methotrexate or surgical treatment (depending on the clinical scenario) [175].
**Uterine perforation**	Suction should be stopped immediately. Evaluate aspirated contents for omentum, bowel, or gestational tissue. If the patient is stable, the procedure may continue under ultrasound guidance, followed by close observation and supportive care with uterotonics and antibiotics. If the patient is unstable, urgent surgical assessment and transfer are required [174,175].
**Vasovagal reaction**	Managed with elevation of the legs above chest level, cool compresses, and isometric extremity contractions. Atropine 0.4 mg IM or 0.2 mg IV may be used as needed (maximum total dose: 2 mg) [175].
**Hemometra (blood accumulation in the uterus)**	May present with abdominal pain, rectal pressure, hypotension, or vasovagal syncope. Management includes uterine aspiration and/or administration of uterotonics [174,175].

**Table 9:** EUROCAT-Reported Congenital Anomalies with Increasing Prevalence (2013–2022).

Increasing Trends Identified at Pan-European Level	
Congenital Anomaly	Annual Change in Prevalence and Possible Explanations
**Double-Outlet Right Ventricle (DORV)**	The annual increase of 3.8% in prevalence (95% CIs: +0.0%; +7.7%) could be attributed to improved diagnostic capabilities. On the other hand, there is a significant variation between different regions, with the lowest prevalence in Vaud (0.35 per 10,000 births) and the highest in Ukraine (2.24 per 10,000 births). An increase has also been observed in certain registries (Saxony-Anhalt, Emilia Romagna and Tuscany) [181,182]. The rising trend could be due to improved coding practices, as this condition was not specifically classified under ICD9, which is still in use in some hospitals.
**Caudal Regression Sequence (CRS)**	The prevalence has increased annually by 16.3% (95% CIs: +0.7%; +34.3%) at the pan-European level, though no registry has reported a significant increase, reflecting its rarity [183,184]. Differences in autopsy rates across registries contribute to variability in reporting. The increase in the rates of obesity and maternal diabetes could represent factors that contribute to these increasing trends
**Patau Syndrome and Edward’s Syndrome**	The prevalence of Patau syndrome has increased by 3.2% annually (95% CIs: +0.4%; +6.2%), while Edward’s syndrome has seen a 2.7% annual increase (95% CIs: +0.5%; +4.8%) [181,183,184]. A possible explanation for these increases may be attributed to broader use of non-invasive prenatal testing, which facilitates earlier detection.

**Table 10:** EUROCAT-Reported Congenital Anomalies with Decreasing Prevalence (2013–2022).

Decreasing Trends Identified at Pan-European Level	
Congenital Anomaly	Annual Change in Prevalence and Possible Explanations
**Hydrocephaly**	Its prevalence has decreased by 3.3% annually (95% CIs: −5.9%; −0.6%). This trend is consistent with previous reports, with significant declines observed in Paris, Réunion, Emilia Romagna and Wielkopolska [181,182,183,184,191]. The decrease may be due to improved maternal health, reduced infections during pregnancy and enhanced prenatal ultrasound screening (which may identify other cerebral anomalies).
**Severe Microcephaly**	Its prevalence decreased by 6.5% annually (95% CIs: −10.1%; −2.8%) [181,182,183,184,191]. This trend might be linked to improved coding practices following the publication of a new guideline on microcephaly by the EUROCAT Coding and Classification Committee in 2016, which likely led to fewer overreporting instances. Significant decreases in prevalence have been noted in certain registries, which may reflect improved classification as well as better prenatal care.
**Anophthalmos**	The prevalence of anophthalmos decreased by 15% from 2013 to 2022 (95% CIs: −24.4%; −4.4%) [183]. On the other hand, due to the rarity of this condition, the estimates are based on small sample sizes, making the trend difficult to interpret. The reasons for this decrease remain unclear, so further surveillance is needed.
**Congenital glaucoma**	The prevalence has decreased by 4.6% annually (95% CIs: −5.0%; −4.2%) between 2013 and 2022 [183]. This decline has been noted in regions such as Réunion and Emilia Romagna, but the reasons remain unclear. Variations in prevalence, such as lower rates in Cork & Kerry and Malta, suggest regional differences in reporting and diagnosis.
**Atrial septal defect (ASD)**	The prevalence of ASD decreased by 5.3% annually (95% CIs: −8.2%; −2.3%) [192]. This decline is considered to be due to less reporting of ASD or persistent foramen ovale cases without follow-up within the first year, as previously reported by EUROCAT. The decrease has been noted across multiple regions and the exact causes remain uncertain.
**Tricuspid atresia and stenosis**	Their prevalence decreased by 6.6% annually (95% CIs: −11.0%; −1.9%) [181,182,183,184,191]. This decrease was observed in one local registry (Paris) and could be attributed to changes in coding practices, with some cases now categorized under hypoplastic right heart syndrome. Further investigation is needed to understand the full reasons for the decline.
**Cleft palate**	It has been observed that its prevalence decreased by 2.7% annually (95% CIs: −4.6%; −0.9%) [182,183]. A significant decline was noted in Wales, with the overall decrease potentially due to an increase in the number of cases with an associated genetic diagnosis. Additional research is needed to better understand this trend.
**Gastroschisis**	The prevalence of gastroschisis decreased by 2.9% annually (95% CIs: −5.6%; −0.1%) [182,183,184,191]. An important decrease was reported in Brittany. It is considered that this trend may be related to fewer teenage pregnancies in some countries (particularly in the UK). However, this decline is now evident across continental Europe, warranting continued monitoring.
**Hypospadias**	The prevalence of hypospadias decreased by 2.2% annually (95% CIs: −3.7%; −0.7%) [182,191]. This decline is reassuring, as previous reports from the 1980s and 1990s showed an increase.
**Pierre Robin sequence**	The prevalence of Pierre Robin sequence decreased by 4.5% annually (95% CIs: −8.2%; −0.6%) [193]. This decrease could be due to less frequent reporting of cases in regions with previously high prevalence, particularly in the French registries.
**Skeletal dysplasias**	The prevalence decreased by 3.1% annually (95% CIs: −5.9%; −0.3%) [182,183]. The reasons for this decrease remain unclear, but the variation in prevalence across registries requires further investigation.

## Data Availability

No new data were created or analyzed in this study.

## Abbreviations

The following abbreviations are used in this manuscript:

ADHD	Attention-deficit/hyperactivity disorder
AFP	Alpha-fetoprotein
AN	Anorexia nervosa
ARPKD	Autosomal recessive polycystic kidney disease
AVSD	Atrioventricular septal defect
BN	Bulimia nervosa
BPD	Borderline personality disorder
bpm	Beats per minute
CAs	Congenital anomalies
CDH	Congenital diaphragmatic hernia
cfDNA	Cell-free fetal DNA
CMA	Chromosomal microarray analysis
CVS	Chorionic villus sampling
DV	Ductus venosus
FISH	Fluorescence in situ hybridization
hCG	Human chorionic gonadotropin
HLHS	Hypoplastic left heart syndrome
IPV	Intimate partner violence
IVF	In vitro fertilization
NIPS	Non-invasive prenatal screening
NT	Nuchal translucency
PGD	Preimplantation genetic diagnosis
PPV	Positive predictive value
PTSD	Post-traumatic stress disorder
STDs	Sexually transmitted diseases
TToP	Therapeutic termination of pregnancy
VToP	Voluntary termination of pregnancy
X-rays	X-radiation
